# Design and Testing of a Separation and Desalination Device for Farmland Saline–Alkaline Water in Arid Areas

**DOI:** 10.3390/ijerph19106178

**Published:** 2022-05-19

**Authors:** Qiaonan Yang, Can Hu, Jie Li, Xiaokang Yi, Jie Zhang, Zhilin Sun

**Affiliations:** 1 College of Mechanical and Electrical Engineering, Tarim University, Alar 843300, China; 10757193082@stumali.taru.cn (Q.Y.); 120140004@taru.edu.cn (C.H.); 10757202220@stumali.taru.cn (J.L.); 10757202212@stumail.taru.edu.cn (J.Z.); 2 Agricultural Engineering Key Laboratory, Universities of Education Department of Xinjiang Uygur Autonomous Region, Tarim University, Alar 843300, China; 3 Ocean College, Zhejiang University, Hangzhou 310058, China; oceansun@zju.edu.cn

**Keywords:** solar-driven seawater desalination, saline–alkaline water, salt leaching through basin irrigation, circulating solar collector tube

## Abstract

To solve the problem of soil salination and to desalinate saline–alkaline water in arid areas, this study involved the design and testing of a separation and desalination device for farmland saline–alkaline water that is suitable for arid areas. The results of this study indicate that after the pretreatment of farmland saline–alkaline water, the water yielded by the pretreatment device had a mean turbidity of <1 and a mean silt density index (SDI) of <3, which met the working conditions of nanofiltration (NF) and reverse osmosis (RO) membranes. When used to filter saline–alkaline water, the composite NF–RO membrane system achieved a desalination rate of 97.06%, a total hardness removal rate of 97.83%, and a Cl^−^ removal rate of 99.65%, which satisfied the standard for irrigation water quality. Some indicators of the yielded water reached the hygienic standard for drinking water, thus successfully reutilizing water resources. The circulating solar collector tube of the device was designed with a collection area of 6 m^2^, which could basically satisfy the heat demand of the flash tank for distillation. The design of the flash tank and the shell-and-tube circulating condenser met the requirements for vapor condensation. The crystals in the solar salt box precipitated under solar action. X-ray diffraction was used to identify the primary compound of the crystals as NaCl, suggesting that the precipitates have potential value as industrial salts. This study offers new technical references and helpful engineering guidance for arid saline–alkaline enrichment areas facing the problem of saline farmland irrigation water.

## 1. Introduction

Soil salination is a major factor that restricts the sustainable development of agriculture in arid areas [1]. It seriously affects the rate of emergence, quality, and yield of crops [2]. Studies indicate that soil salination has affected 75 countries worldwide, resulting in an overall saline–alkaline land scale of approximately 9.5 × 10^7^ hm^2^ [3]. In addition, a total area of 7.7 × 10^7^ hm^2^ of land suffers from secondary salination [4]. The Xinjiang Uygur Autonomous Region (hereafter referred to as “Xinjiang”) accounts for 22.01% [5] of the total area of China’s saline–alkaline lands, with the widest distribution of saline–alkaline lands [6] and the highest degree of soil salinity in China. Soil salination has directly harmed the sustainable development of the regional economy in Xinjiang [7,8]. To improve agricultural productivity and ensure grain security in arid areas [9,10], farmers have extensively adopted the technique of salt leaching through basin irrigation to actively minimize soil salination [11]. This technique involves irrigating farmlands to dissolve the soil salts and discharge soluble salts from the topsoil into deep soil (or leach them out) through infiltration. In this way, harmful soil contents in the soil are reduced through lateral seepage into drainage canals, thus making the soil arable. However, this measure makes it difficult to desalinate the saline–alkaline water discharged after salt leaching. Currently, soil salts are mainly discharged into rivers and agricultural areas through farmland alkali drainage canals, thereby intensifying soil salination and impacting ecological environments on larger scales. Drinking saline–alkaline water on a long-term basis [12] causes diseases, such as hypertension and angiocardiopathy, and it harms the health of people who live in such areas. In this context, the key to fundamentally preventing and governing soil salination lies in efficiently separating and desalinating the agricultural saline–alkaline water discharged after salt leaching through basin irrigation.

There is an urgent need for an effective separation and desalination technique in order to separate and desalinate farmland saline–alkaline water in arid areas. Existing studies mainly focused on techniques related to the desalination of seawater and brackish water, but rarely touched upon farmland saline–alkaline water in arid areas. In 1988, Tiwari et al. [13] was the first to explore the factors that influence the design and working conditions of cross-tube solar stills. They found that the water yield of cross-tube solar stills was higher than that of traditional disc-type solar stills under the same weather conditions. In 2000, Dai, Y.J. et al. [14] designed a solar seawater desalination device with humidification–dehumidification (HDH) functions. The device used a falling-film humidifier with an evaporation surface and forced convection to enhance heat and mass transfer. By mounting a sprayer in the humidifier to increase evaporation efficiency, the device achieved a thermal efficiency that was greater than 80%. In 2008, Yang [15] built a solar membrane distillation test bench with a collector area of 8 m^2^ in Hangzhou and achieved a water yield of 954 L/(y·m^2^) using the solar collector. In 2010, Cho, Y. et al. [16] recorded the solar irradiation quantities on the inclined surfaces of solar collectors in Daejeon, South Korea, based on inclination data and compared each result with the result estimated with the proposed solar irradiation formula. In 2012, Zhang et al. [17] designed a special multiple-effect evaporation zone during vacuum membrane distillation (VMD) and endowed the membrane module with both heat exchange and cooling functions of vapor and the heating and evaporation functions of a feed solution, thus efficiently recycling the latent heat of evaporation in the VMD. In 2013, Martinopoulos, G. et al. [18] investigated the performance characteristics of flat-plate solar collectors using flow data. In 2017, Liu, J. et al. [19] developed a small-sized solar seawater desalination device and obtained the maximum freshwater yield of each season by changing the mounting angle of the solar collector. They found that the maximum freshwater yields of spring, summer, fall, and winter were achieved at mounting angles of 300, 150, 300, and 450, respectively. In 2018, Liu et al. [20] designed a solar VMD-RO concentrated water desalination system suitable for cold areas and discovered that the freshwater yield declined with intensifying membrane fouling and increased 1.5 fold after membrane cleaning. In 2019, Lacroix et al. [21] designed an innovative thermal-drive reverse osmosis seawater desalination device. This new device used solar energy to pressurize seawater and make it exceed the osmotic pressure of the device in order to perform seawater desalination. In 2020, Bdour et al. [22] designed an off-grid photovoltaic-driven reverse osmosis membrane desalination device in which the emphasis was on reducing the storage capacity required for normal operation of the device under specific requirements. The results showed that controlling inlet water pressure could improve the separation efficiency and reduce energy consumption. In 2021, Zhang [23] proposed a new process that combined the traditional thermal method and membrane separation method; this process used vacuum membrane distillation for a seawater desalination test, and the influences of temperature, vacuum degree, flow rate, inlet water quality, and other factors on membrane flux and water TDS were investigated. The enhancement effect of gas–liquid two-phase flow on membrane flux was studied. The pretreatment and membrane-cleaning processes of calcium and magnesium were put forward. In 2022, Ali [24] optimized the control system of an intermittent wind-driven brackish water reverse osmosis desalination device, which continuously adjusted the reverse osmosis desalination device through the auxiliary wind-driven water storage tank. In summary, the separation and desalination of farmland saline–alkaline water are still in the exploratory stage, and efforts are still needed to develop an efficient and effective separation and desalination technique for saline–alkaline water.

The authors of this study designed a solar separation and desalination device for farmland saline–alkaline water in arid areas, tested it at the Soil Tank Laboratory of Tarim University, Alar City, Xinjiang, and validated the rationality of its design. This study offers new technical references for solving the problem of farmland irrigation water faced in arid saline–alkaline areas.

## 2. Composition, Working Principle, and Design

### 2.1. Composition

**Figure 1:**
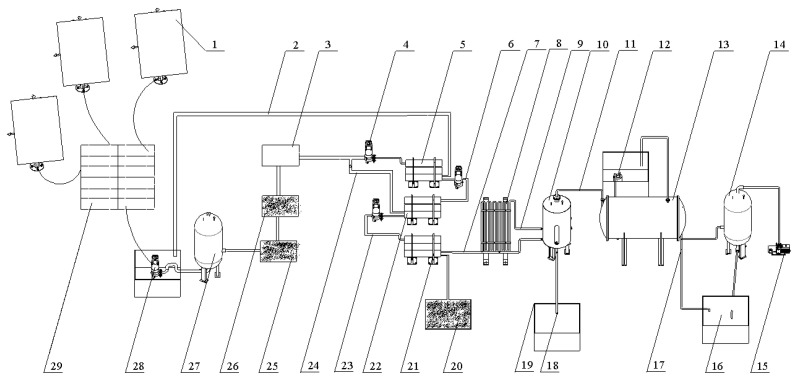
Schematic diagram of the farmland saline–alkali water separation and desalination device: (1) photovoltaic panel; (2) primary reverse osmosis concentrated water outlet; (3) ultrafiltration membrane; (4) variable pressure pump; (5) primary reverse osmosis membrane; (6) primary outlet for water produced through reverse osmosis; (7) tertiary nanofiltration concentrated water outlet; (8) solar cycle collector; (9) concentrated water inlet of the flash tank; (10) flash tank; (11) steam outlet; (12) water circulating pump; (13) shell-and-tube circulating condenser; (14) negative pressure tank; (15) vacuum pump; (16) freshwater tank; (17) fresh water export; (18) salt outlet of the flash tank; (19) salt-drying box; (20) farmland; (21) tertiary nanofiltration membrane; (22) secondary reverse osmosis membrane; (23) secondary outlet for water produced through reverse osmosis; (24) secondary reverse osmosis concentrated water outlet; (25) graphite carbon filter; (26) quartz sand filter; (27) towerless water supply; (28) submersible pump; (29) battery.

The separation and desalination device designed in this study was mainly composed of a pretreatment device, which further consisted of an activated carbon filter, a quartz sand filter, an ultrafiltration filter, a composite NF–RO membrane system, a circulating solar collector tube, a flash tank, a shell-and-tube circulating condenser, a negative pressure chamber, a vacuum pump, a water tank, and a solar salt box, as illustrated in Figure 1. The device was installed at the Soil Tank Laboratory of Tarim University, Alar City, Xinjiang, China. The solar photovoltaic power generation system consisted of 32,350 W photovoltaic panels, 16,250 Ah12 V batteries, and 20,000 W power frequency inverters. The daily power generation was 44 degrees, and the device consumed 3.5 degrees of electricity per hour. The device ran for 11 h at each time, and the power generation met the daily electricity demand of the installation. A composite NF–RO membrane system used for farmland irrigation was located on the front end of the device. Considering the unavailability of the concentrated water obtained through filtration by the composite NF–RO membrane system, a flash tank was mounted for distillation and desalination. The concentrated water that flowed into the flash tank was heated by a circulating solar collector tube to reduce energy consumption. The vapor generated through distillation by the flash tank was cooled using a shell-and-tube circulating condenser.

### 2.2. Working Principle

As illustrated in Figure 1, farmland saline–alkaline water was pumped from the water tank into a towerless water supply device, pressurized there, and successively led through the quartz sand filter, the activated carbon filter, and the ultrafiltration filter for pretreatment. The water yielded after pretreatment was then driven by a high-pressure pump into the composite NF–RO membrane system. The freshwater yielded was used to irrigate farmlands, while the strong brine flowed into the strong brine tank. After being heated by the circulating solar collector tube, the strong brine flowed into the flash tank. The vapor generated through the distillation of the strong brine was pumped by the vacuum pump into the shell-and-tube circulating condenser and condensed by a low-temperature strong brine. The freshwater obtained through condensation was used to irrigate the farmland, thus reutilizing water resources. The heat in the shell-and-tube circulating condenser was used to preheat low-temperature saline–alkaline water. The strong brine in the flash tank turned into a concentrate after repeated distillation and was ultimately discharged into the solar salt box for solar evaporative crystallization.

### 2.3. Design of Key Parts

#### 2.3.1. Pretreatment Modules

The pretreatment device further consisted of an activated carbon filter, a quartz sand filter, and an ultrafiltration filter. In particular, the activated carbon filter and the quartz sand filter were developed by this laboratory. They had a working pressure of 0.3–0.4 MPa and a water yield of 2.5 m^3^/h. The activated carbon filter (600 mm in diameter, 1600 mm high, and 1200 mm in layer height) was used to filter particles of 1.0~1.5 mm in size. It could intercept large particle impurities and effectively remove suspended solids, colloids, and sediments from saline–alkaline water. The quartz sand filter (600 m in diameter, 1600 mm high, and 1200 mm in layer height) was used to filter particles of 0.8~1.0 mm. It could adsorb peculiar colors and smells and reduce the turbidity, residual chlorine, and pesticide residues of saline–alkaline water. The ultrafiltration filter (Model RM-UF-4046W, China Jiangsu Suzhou Run membrane water treatment technology Co., Ltd., Suzhou, China) was made of PVDF material, the effective membrane area was 7.0 m^2^, the inner diameter of the membrane wire was 0.7 mm, the outer diameter was 1.3 mm, the design flux was 50–100 L/h/m^2^, and the intercepted molecular weight was 100,000. The ultrafiltration filter could remove fine sediments and other impurities from the saline–alkaline water; it clogged infrequently, was easily cleaned, and did not produce any wastewater.

#### 2.3.2. Filter Modules

The filtration equipment of the farmland saline–alkaline water separation and desalination device was composed of a nanofiltration–reverse osmosis membrane (nanofiltration membrane model RM-NF-4040, reverse osmosis membrane model RM-BW-4040, Jiangsu Suzhou Run Membrane Water Treatment Technology Co., Ltd., Suzhou, China), and the nanofiltration–reverse osmosis membrane was made of aromatic polyamide composite membrane material; the sludge density index of the inlet water quality was <5, and the turbidity was <1. The working pressure of the nanofiltration membrane was 0.48 Mpa, the effective membrane area was 3.3 m^2^, the average water permeability was 7.9 m^3^/d, the aperture was 1 nm, and the molecular weight of about 200–800 organic matter could be retained. The working pressure of the reverse osmosis membrane was 1.55 Mpa, the effective film area was 7.9 m^2^, the average water permeability was 7.2 m^3^/d, and all dissolved salts and molecular weights could be retained with >100 organic matter.

#### 2.3.3. Pretreatment Modules

In general, it is very difficult for ordinary flat-plate or tubular solar collectors to heat concentrated water to 80 °C. We improved the integrated heat absorption–evaporation circulating solar collector tube proposed by Sun et al. [25] to increase the efficiency of solar collectors. The collector tube was made of quartz tubes to avoid the corrosion of the circulating solar collector tube by the concentrated water. Its highly transparent outer tube was 58 mm in diameter, 4 mm thick, and 1600 mm high. Its inner tube was 30 mm in diameter, 4 mm thick, 1600 mm high, and coated with a blue film that strongly absorbed light. A vacuum was reserved between the inner and outer tubes to enhance the absorption of light and heat and to thermally insulate the circulating solar collector tube. The collector tube was assembled using U-type series connections to continuously heat the concentrated water that entered the collector tube. The installation angle was geared to the local latitude of Alar City. The maximum working pressure was set to 0.6 MPa. The concentrated water that entered the circulating solar collector tube was heated to 80 °C. The temperature of the concentrated water generated by the composite NF–RO membrane system was 25 °C. The required circulating solar collector tube area was *A_C_* [26], which was calculated from Equation (1):

(1) $$A_{C}=\frac{Q_{W}c\rho \left(t_{end}-t_{L}\right)f}{J_{T}\eta_{cd}\left(1-\eta_{L}\right)}$$

where *Q_W_* denotes the mean daily hot water capacity, m^3^; *c* denotes the specific heat at constant pressure of the concentrated water in KJ/(kg. °C); ρ denotes the density of the concentrated water in kg/m^3^; *t_end_* denotes the designed heating temperature of the circulating solar collector tube in °C; $t_{L}$ denotes the temperature of the concentrated water generated by the composite NF–RO membrane system in °C; *J_T_* denotes the mean annual solar irradiation on the plane of installation inclination in Alar City [26] in MJ/m^2^; *f* denotes the solar fraction, set as 60% [26]; $\eta_{cd}$ denotes the all-day heat-collecting efficiency of the solar collector, set as 72.7%; $\eta_{L}$ denotes the heat-loss rate of the circulating solar collector tube, set as 20%. Based on the results of calculations of Equation (1), the collector area required to heat the concentrated water in the circulating solar collector tube to the set temperature was 5.72 m^2^.

#### 2.3.4. Flash Tank

A flash tank was adopted with a vertical split design [27], and it was constructed from 6-mm-thick Q235. The designed water yield of the flash tank was 50 L/h. The temperature of the concentrated water entering the flash tank was 80 °C. The vacuum degree was 0.85 bar.

(1) Flow velocity of the gas

The flow velocity of the gas generated by the 80 °C concentrated water through flash in the flash tank was $u_{G}$ [27], as expressed by Equation (2):

(2) $$u_{G}=K_{G}{\left(\frac{\rho_{L}-\rho_{G}}{\rho_{G}}\right)}^{0.5}$$

where $K_{G}$ denotes the coefficient of the flow velocity of the gas; $\rho_{L}$ denotes the density of the 80 °C concentrated water in kg/m^3^; $\rho_{G}$ denotes the density of the 80 °C vapor in kg/m^3^. The flow velocity of the gas in the flash tank was calculated to be 2.95 m/s using Equation (2).

(2) Diameter of the flash tank

The diameter of the flash tank was D [27], as expressed by Equation (3):

(3) $$D=\omega {\left(\frac{\varphi V_{G}}{u_{G}}\right)}^{0.5}$$

where *ω* is a constant, set as 0.0188; *φ* denotes the maximum coefficient of the volumetric flow rate; $V_{G}$ denotes the volumetric flow rate of the gas in m^3^/h; $u_{G}$ denotes the flow velocity of the vapor in the flash tank in m/s. The diameter of the flash tank was calculated to be 456 mm (500 after rounding) using Equation (3).

(3) Height of the flash tank

The height of the flash tank was *H* [27], as expressed by Equation (4):

(4) $$H=H_{L}+2H_{d}$$

where $H_{L}$, the simplified height of the flash tank, in mm, can be obtained based on the diameter D of the flash tank in accordance with the Specification for Oil and Gas Separators (SYT0515-2007) [28], and $H_{d}$ (i.e., the height of the head, in mm) can be obtained from D/4 in accordance with the Formed Heads for Steel Pressure Vessels (JB/T4746-2002) [29]. The total height of the flash tank was calculated to be 1800 mm using Equation (4).

#### 2.3.5. Shell-and-Tube Circulating Condenser

The shell-and-tube circulating condenser was constructed from heat exchange tubes that were 25 mm in diameter and 2.5 mm thick.

(1) Calculation of heat release

The heat released by the 80 °C vapor through condensation into freshwater (assuming that the mean temperature was 20 °C) was *Q* [30], as expressed by Equation (5):

(5) $$Q=W_{c}W_{pc}\left(t_{2}-t_{1}\right)$$

where $W_{c}$ is the mass flow rate of the fluid in kg/h; $C_{pc}$ is the average constant-pressure specific heat capacity of the fluid in kJ/(kg·°C); *t* is the fluid temperature in °C.

(2) Number of stainless-steel radiating tubes

The number of stainless-steel radiating tubes was *N* [30], as described by Equation (6):

(6) $$N=\frac{m/\rho }{\frac{\pi }{4}d_{i}^{2}u_{i}}$$

where ρ denotes the density of the cooling water in kg/m^3^; $d_{i}$ denotes the inner diameter of the stainless-steel radiating tubes in m; $u_{i}$ denotes the flow velocity of the cooling water in m/s. The number of stainless-steel radiating tubes was calculated to be 145.1 (146 after rounding) with Equation (6).

(3) Length of the stainless-steel radiating tubes

The length of the stainless-steel radiating tubes was *L* [30], as expressed by Formula (7):

(7) $$L=\alpha S/N\pi d_{0}$$

where α denotes the area margin constant; *S* denotes the heat transfer area in m^2^; $d_{0}$ denotes the diameter of the stainless-steel radiating tubes in m. The length of the stainless-steel radiating tubes was calculated to be 2.52 m (3 m after rounding) using Equation (7).

(4) Diameter of the shell-and-tube circulating condenser

The diameter of the shell-and-tube circulating condenser was *D* [30], as expressed by Equation (8):

(8) $$D=1.05t\sqrt{N/\eta }$$

where *t* denotes the tube pitch in mm, and *η* denotes the plate and tube utilization rate, set as 70%. The inner diameter of the shell-and-tube circulating condenser was calculated to be 485 mm (500 mm after rounding) with Equation (8).

## 3. Materials and Methods

### 3.1. Source and Composition of Saline–Alkaline Water

**Figure 2:**
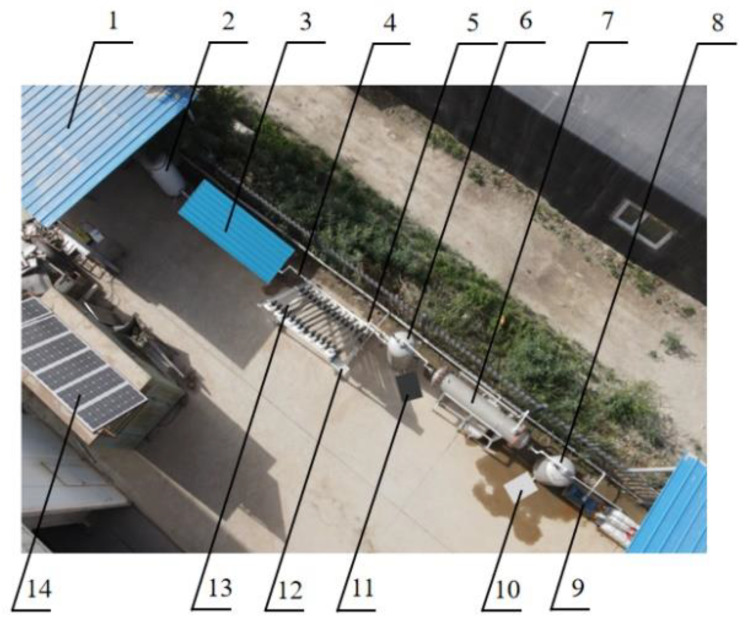
Physical drawing of the farmland saline–alkali water separation and desalination device: (1) shading canopy; (2) towerless water supply; (3) pretreatment filtration equipment; (4) inlet of the solar cycle collector; (5) flash drum inlet; (6) flash tank; (7) shell-and-tube circulating condenser; (8) negative pressure tank; (9) water ring vacuum pump; (10) freshwater tank; (11) salt-drying box; (12) control box; (13) solar cycle collector; (14) photovoltaic panel.

The saline–alkaline water under investigation in this study was sampled from the #10 alkali drainage canal of Alar City, Xinjiang, China (40°39′26.21″ N, 81°23′28.69″ E; 1012 m in elevation) in June 2021, 45 d after spring irrigation. The saline–alkaline water collected from the sampling site was protected from direct sunshine under a sunshade on the test site. The test site was located at the Soil Tank Laboratory of Tarim University, Alar City, Xinjiang, China, as shown in Figure 2 [31]. The test time was from 9:00 to 20:00 every day, working continuously for 8 days (from 9 June 2021 to 16 June 2021), collecting 1000 L of water samples every day. The collected saline–alkaline water sample had a mean salinity of 16,834 mg/L, a mean turbidity of 6.9 NTU, and a mean silt density index (SDI) of 6.3. Table 1 provides the composition of the saline–alkaline water after pretreatment.

### 3.2. Test Design

#### 3.2.1. Pretreatment Effect Test

Farmland saline–alkaline water was successively led through the quartz sand filter, the activated carbon filter, and the ultrafiltration filter to keep the pretreatment device at a stable pressure. The salinity, turbidity, and SDI of the water yielded were measured to analyze the pretreatment effect of the pretreatment device.

#### 3.2.2. Comparison of the NF Membrane, the RO Membrane, and the Composite NF–RO Membrane System in Terms of the Filtering Effect

To keep the NF and RO membranes under stable working pressures, the filtration membranes of the separation and desalination device for farmland saline–alkaline water were combined in different manners to compare the NF membrane, the RO membrane, and the composite NF–RO membrane system in terms of the filtering effect. The effects of the filter unit on the salinity, total hardness, cation content, and anion content of the water yielded through the outlet of each membrane were analyzed.

#### 3.2.3. Heat-Collecting Effect of the Circulating Solar Collector Tube

The heat-collecting effect of the circulating solar collector tube was analyzed by conducting continuous tests on local solar irradiance, ambient temperature, ambient humidity, and the inlet and outlet temperatures of the circulating solar collector tube.

#### 3.2.4. Distillation Test of the Flash Tank

(1) To keep the vacuum degree of the flash tank constant, the temperature of the concentrated water at the inlet of the flash tank was changed to analyze how the temperature affected the flash effect.
(2) To ensure that the temperature of the concentrated water entering the flash tank remained constant, the vacuum degree of the flash tank was changed to analyze how the vacuum degree affected the flash effect.

#### 3.2.5. Condensation Effect of the Shell-and-Tube Circulating Condenser

The inlet and outlet temperatures of the shell-and-tube circulating condenser were monitored to measure the drop in temperature of the vapor in the radiating tubes and to further analyze the condensation effect of the shell-and-tube circulating condenser.

#### 3.2.6. Solar Salt-Making Test

After continuously absorbing solar irradiation, the concentrated water in the solar salt box gradually evaporated and finally crystallized. The precipitation rates and components of the crystals were analyzed to determine their values.

### 3.3. Test Indices and Methods

#### 3.3.1. Indices and Methods of the Pretreatment Test

The test indicators of the pretreatment effect included the salt content, turbidity, and silt density index of the farmland saline–alkali water before and after pretreatment. ① Salinity of saline–alkali water: The AR8012 pen-type salinity meter (Instruments Co., Ltd., Dongguan, China) was used to detect the salinity of saline–alkali water in the farmland before and after pretreatment. ② Turbidity of saline–alkali water: The SIN-PTU100 industrial turbidity meter (Sinomeasure Automation Technology Co., Ltd., Hangzhou, China) was used to detect the turbidity of farmland saline–alkali water before and after pretreatment. ③ Density index tester for saline–alkali water (SDI): The QZDY-SD147 silt density index (SDI) tester (Qibote Environmental Science and Technology Co., Ltd., Wuxi, China) was used to detect the silt density index (SDI) of farmland saline–alkali water before and after pretreatment.

#### 3.3.2. Filter Effect Test Index and Method for the Membrane Combination Comparison

The indices used to evaluate the separation and desalination effect of the composite NF–RO membrane system included the desalination rate, total hardness, and Cl^−^ content.

① Desalination rate: The salinity of the original saline–alkaline water, the water yielded by the primary and secondary RO membranes, and the water yielded by the tertiary NF membrane were measured using a pen-type salimeter (model: AR8012; salinity measurement range: 0–50 ppt; resolution: 0.01 ppt; Guangdong Smart Instruments Co., Ltd., Dongguan, China). The desalination rate is *R*, as expressed by Equation (9):

(9) $$R=\left(1-\frac{C_{p}}{C_{f}}\right)\times 100\%$$

where *R* is the rejection rate in %; $C_{p}$ is the salt content in the produced water in mg/L; $C_{f}$ is the salt content in the water sample in mg/L.

② Total hardness: EDTA titration was used to measure the total hardness of the original saline–alkaline water, the water yielded by the primary and secondary RO membranes, and the water yielded by the tertiary NF membrane. The total hardness was *Z*, as expressed by Equation (10):

(10) $$Z=\beta \times \frac{V_{1}\times G}{V}$$

where β is a constant of 1000; $V_{1}$ is the number of milliliters of EDTA standard solution consumed during titration in mL; *G* is the equivalent concentration of the EDTA standard solution in mol/L; *V* is the volume of the water sample in milliliters.

③ Cl^−^ content: The Cl^−^ content of the original saline–alkaline water, the water yielded by the primary and secondary RO membranes, and that of the water yielded by the tertiary NF membrane was measured using an ion chromatograph (model: Integrion; chromatographic column: AS11-HC-4; intelligent high-pressure ion chromatograph manufactured by Thermo Fisher Scientific Inc.). The Cl^−^ removal rate is *P*, as expressed by Equation (11):

(11) $$P=\frac{\rho_{c1}-\rho_{c2}}{\rho_{1}}\times 100\%$$

where $\rho_{c1}$ is the Cl^−^ content in the original water sample in mg/L; $\rho_{c2}$ is the Cl^−^ content in the produced water in mg/L.

#### 3.3.3. Filter Effect Test Index and Method for the Membrane Combination Comparison

The indices of the heat-collecting effect included the local solar irradiance, ambient temperature, ambient humidity, and the inlet and outlet temperatures of the circulating solar collector tube. ① Local solar irradiance, ambient temperature, and ambient humidity were monitored using RS485 illuminance, temperature, and humidity sensors (SXN Electronics Technology Co., Ltd., Jinan, China). The range of illuminance that was measured was 0~65,535 Lux. The range of temperatures was −40~60 °C. The range of humidity that was measured varied from 0% RH to ~80% RH. The precision of the measurements was as follows: illuminance intensity ≤ 5%/y, temperature ≤ 0.1 °C/y, and humidity ≤ 1%/y. The heat-collection efficiency *η* of the solar circulation heat-collector tube is expressed by Equation (12):

(12) $$\eta =\frac{Q_{FN}}{IA_{FN}}$$

In Equation (12), $Q_{FN}$ is the total heat energy absorbed by the solar circulation heat-collecting tube in J; *I* is the total solar radiance in W/m^2^; $A_{FN}$ is the heat absorption area of the inner tube in m^2^.

② The inlet and outlet temperatures of the circulating solar collector tube were monitored using DS18b20 temperature sensors (Senxte Co., Ltd., Changzhou, China). The temperatures that could be measured ranged from −55~+125 °C, and the precision of the measurements ranged from 9 to 12 Bit.

#### 3.3.4. Indices and Methods of the Flash Effect Test

The flash effect was evaluated using the indices of vacuum degree and temperature. ① The vacuum degree of the flash tank was measured using a PT210B-M20 pressure sensor (Yunhao Automation Equipment Co., Ltd., Shanghai, China) that measured a range of pressure from −0.1 to 2 MPa with a precision of 0.5% FS. The pump was a 5.5 kW water-ring vacuum pump with a maximum suction intensity of 230 m^3^/h and an ultimate vacuum degree of 0.97 bar. ② The temperature in the flash tank was measured using a DS18b20 temperature sensor (Senxte Co., Ltd., Shanghai, China).

#### 3.3.5. Indices and Methods of the Condensation Effect Test

The decrease in temperature and the freshwater recovery rate were adopted to evaluate the condensation effect. ① The inlet and outlet temperatures of the shell-and-tube circulating condenser were monitored using DS18b20 temperature sensors (Senxte Co., Ltd.). ② Freshwater recovery rate: The flow rate through the inlet of the flash tank, the salt outlet of the flash tank, and the freshwater outlet of the shell-and-tube circulating condenser were measured using HI2560 flow sensors (Ponai Sensor Science and Technology Co., Ltd., Leqing, China) that could measure flow rates ranging from 4 to 45 L/min and had a pile-up pulse of 1 L = 225 Hz ± 10%. The quantity of concentrated water that flowed from the circulating solar collector tube to the flash tank and out of the salt outlet of the flash tank was recorded along with the total quantity of the freshwater that flowed out of the freshwater outlet of the shell-and-tube circulating condenser. The freshwater recovery rate was calculated in the end.

#### 3.3.6. Test Indicators and Methods for Solar Salt Drying

The indices used to evaluate the effect of solar salt making through brine evaporation in the sun included the crystallization rate and crystal components. ① Crystallization rate: Crystals were weighed using an electronic balance (model: Pioneer CP series; range: 0–3200 g; measurement precision: 0.001 g; manufactured by Ohaus Instruments (Jiangsu) Co., Ltd., Changzhou, China), and relevant data were recorded. The crystal recovery rate is *Y*, as expressed by Equation (13):

(13) $$Y=\frac{WR\left[C_{1}-C_{2}\left(1-V\right)\right]}{1-C_{2}\left(R-1\right)}$$

where $C_{1}$ and $C_{2}$ are the concentrations of the concentrated water and concentrated water compounds, respectively, in mol/L; *V* is the evaporation of concentrated water in L; *R* is the relative molecular mass ratio of concentrated water compounds to concentrated water; W is the salt concentration in concentrated water in mg/L.

② Crystal components: Crystal components were detected using the X-ray diffraction (XRD) method with an X-ray diffractometer (model: D8ADVANCE, manufactured by Bruker (Germany); working voltage: 45 kV; working current: 10 mA; continuous scanning angle: 4–80°).

### 3.4. Date Processing and Analysis

Excel 2007 software manufactured by Microsoft Co., Ltd., Raymond, WA, USA) was used for statistical processing of the data obtained in the test. The standard error S was adopted as the water sample’s salinity error.

(14) $$S=\pm \sqrt{\frac{\sum_{i=1}^{n}{\left(x_{i}-\bar{x}\right)}^{2}}{n-1}}$$

where $x_{i}$ is the salinity, $\bar{x}\;$ is the mean value, and n is the number of sampling times.

## 4. Results and Analysis

### 4.1. Effect of Pretreatment

The collected saline–alkaline water sample had a mean salinity of 16,834 mg/L with a salinity error of S (±0.34 mg/L). After pretreatment, the saline–alkaline water experienced a reduction of 4.3% in mean salinity and had a turbidity of <1 NTU and an SDI of <3, which met the working conditions of the composite NF–RO membrane system. Table 2 provides detailed test data.

### 4.2. Filtration Effects of the NF Membrane, RO Membrane, and Composite NF–RO Membrane System

The pretreated saline–alkaline water had a mean salinity of 16,110 mg/L, a mean total hardness of 1196.4 mg/L, and a mean Cl^−^ content of 7468.89 mg/L. The water yielded after pretreatment was treated further by a filter unit. When the RO membrane alone was used on the filter unit in the test, the desalination rate, total hardness removal rate, and Cl^−^ removal rate were 93.01%, 96.69%, and 96.74%, respectively. The final water yielded had a mean salinity of 1165.53 mg/L, a mean total hardness of 28.23 mg/L, and a mean Cl^−^ content of 509.82 mg/L. When the NF membrane alone was used, the desalination rate, total hardness removal rate, and Cl^−^ removal rate were 94.83%, 97.94%, and 96.83%, respectively. The final water yielded had a mean salinity of 985.97 mg/L, a mean total hardness of 22.01 mg/L, and a mean Cl^−^ content of 182.24 mg/L. When the composite NF–RO membrane system was used, the desalination rate, total hardness removal rate, and Cl^−^ removal rate were 97.06%, 97.83%, and 99.65%, respectively. The final water yielded had a mean salinity of 631.66 mg/L, a mean total hardness of 12.8 mg/L, and a mean Cl^−^ content of 50.79 mg/L.

As shown in Table 3, when the RO membrane alone was used for filtration on the filter unit of the separation and desalination device, it failed to meet the requirements of the Standard for Irrigation Water Quality (GB 5084-2021) (non-saline–alkaline areas: salinity ≤ 1000 mg/L; saline–alkaline areas: salinity ≤ 2000 mg/L, C1^−^ ≤ 350 mg/L) [32]. However, the NF membrane alone could meet the requirements of this document. Notably, the composite NF–RO membrane system filtered more effectively than the NF membrane and satisfied the process demands of the front-end technology of the separation and desalination device for farmland saline–alkaline water.

### 4.3. Heat-Collecting Effect of the Circulating Solar Collector Tube

**Figure 3:**
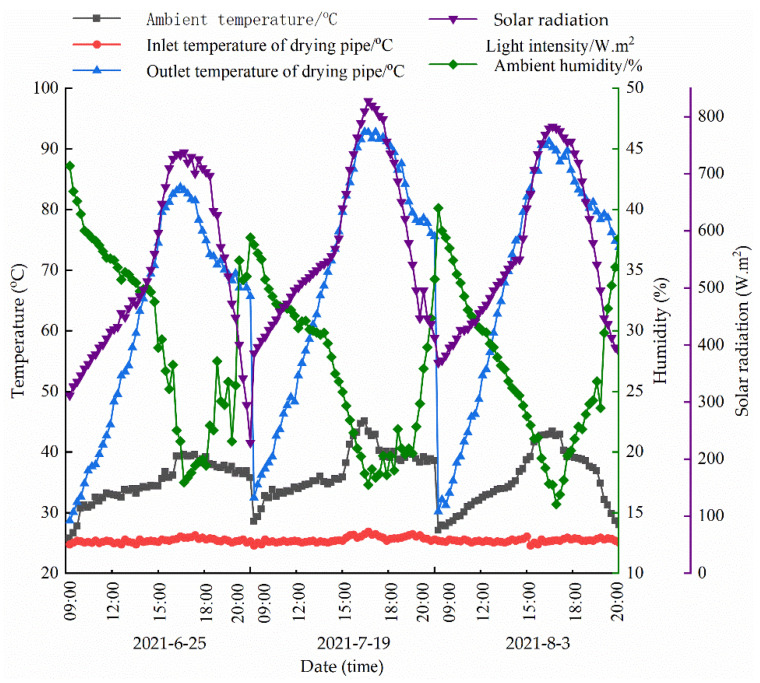
Heat-collection effect of the solar-cycle heat-collection tube.

The circulating solar collector tube was tested from June to August 2021. Typical days were selected in June, July, and August for data analysis. Figure 3 shows the variation curves of the circulating solar collector tube on 25 June, 19 July, and 3 August.

As shown in Figure 3, in June, the circulating solar collector tube could heat the concentrated water filtered by the composite NF–RO membrane system to approximately 85 °C, while it could heat the concentrated water to approximately 90 °C in July and August. Clearly, the heat demand of the flash tank for distillation could be satisfied in June, July, and August owing to the intense solar irradiation and high ambient temperatures.

### 4.4. Analysis of the Effect of Flash Tank Distillation

**Figure 4:**
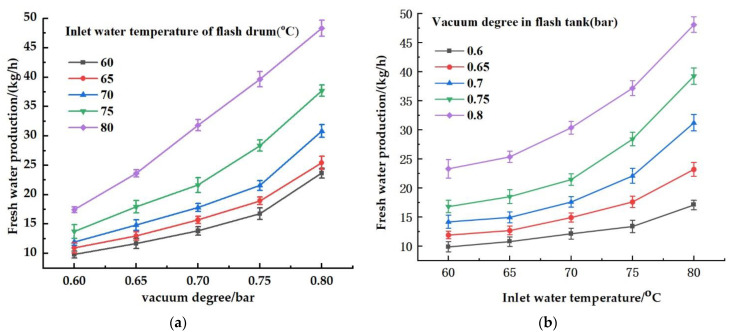
Effects of different temperatures and vacuums on freshwater output in the flash tank: (**a**) Inlet water temperature of the flash drum; (**b**) vacuum degree in the flash tank.

As shown in Figure 4a, the test was conducted when the ambient temperature was above 25 °C. The salinity degree of the concentrated water that was filtered by the composite NF–RO membrane system was 9.6 g/L, and the flow rate through the inlet of the flash tank was 1 m^3^/h. When the vacuum degree of the flash tank was maintained at 0.6, 0.65, 0.7, 0.75, and 0.8 bar under these conditions, the freshwater yield increased with the increasing inlet temperature of the concentrated water. When the vacuum degree was 0.8 bar, the freshwater yield at 80 °C was 1.57 times greater than that at 70 °C and 2.04 times greater than that at 60 °C. As shown in Figure 4b, when the inlet temperature of the concentrated water was maintained at 60, 65, 70, 75, and 80 °C under the same conditions, the freshwater yield increased in parallel with the vacuum degree of the flash tank. When the inlet temperature of the concentrated water was 80 °C, the freshwater yield at 0.8 bar increased by approximately 1.2, 1.5, 2, and 2.8 times on the basis of that at 0.75, 0.7, 0.65, and 0.6 bar, respectively. Thus, the freshwater yield of the flash tank increased with the increasing inlet temperature of the concentrated water and with the increasing vacuum degree of the flash tank, as expressed by the functional relationship [33] in Equation (15).

(15) $$U=\gamma \left(P_{m}-P_{v}\right)$$

where *U* denotes the coefficient of flash; $P_{m}$ denotes the pressure of the vapor in the flash tank in Pa; $P_{v}$ denotes the pressure of the vacuum pump in Pa.

The vapor pressure can be calculated from the Antoine formula [34], as expressed in Equation (16).

(16) $$\log p=A-\frac{B}{T+C}$$

where *A*, *B*, and *C* are all constants (for water, *A* = 16.38, *B* = 3885.70, *C* = 230.70) [34], and *T* denotes the temperature of the vapor in °C.

### 4.5. Condensation Effect of the Shell-and-Tube Circulating Condenser

**Figure 5:**
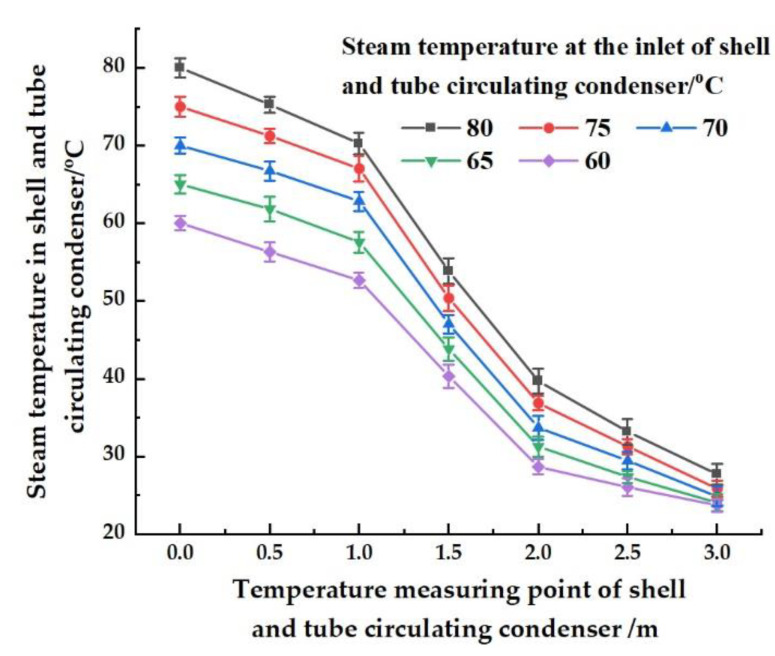
Temperature drop effect of the shell-and-tube circulating condenser.

As shown in Figure 5, the inlet and outlet of the shell-and-tube circulating condenser were taken as the origin and endpoint of the x-coordinate. There was a distance of 3 m between the inlet and outlet. The temperatures of the radiating tubes at 0, 1.0, 2.0, and 3.0 m away from the inlet were measured. When the temperature of the vapor at the inlet was 80, 75, 70, 65, and 60, the vapor flowed in heat exchange tubes and approached the temperature of the cooling water (approximately 25 °C) at the outlet. This meant that the design of the shell-and-tube circulating condenser could meet the requirements of vapor condensation.

### 4.6. Effect of Solar Salt Making

#### 4.6.1. Precipitation Rates of Crystals Formed by Concentrated Water in the Solar Salt Box

**Figure 6:**
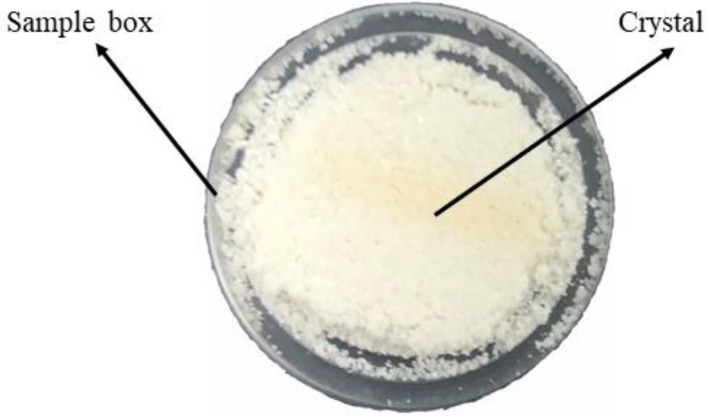
Concentrated water crystals after drying of the salt.

The concentrated water flowing out of the salt outlet of the flash tank entered the solar salt box, continuously evaporated, and was condensed through the absorption of solar irradiation, resulting in the formation of crystals, as shown in Figure 6. Continuous solar salt was prepared from 9 June to 16 June 2021. Table 4 provides the detailed test data, where “a” is the theoretical crystallization weight (kg) and “b” is the actual crystallization weight (kg). During the crystallization process of the concentrated water, the highest, lowest, and mean precipitation rates of crystals were 98.13%, 95.94%, and >95%, respectively.

#### 4.6.2. Components of Crystals in the Solar Salt Box

**Figure 7:**
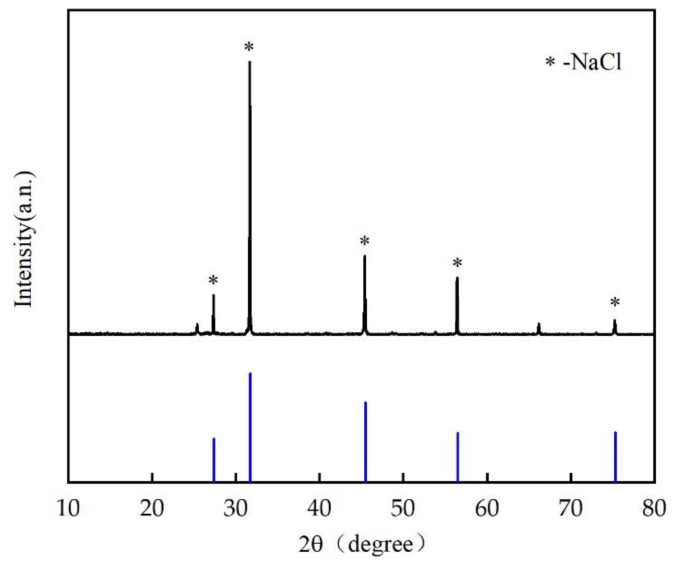
X-ray diffraction pattern of crystals in the salt-drying box.

Figure 7 shows the X-ray diffraction (XRD) diagram of the crystallization of the concentrated water. Clearly, the comparison between the XRD data and the PDF standard card produced a good peak goodness of fit. The primary compound in the crystals was NaCl. Table 5 provides an analysis of the components of the crystals using an energy-dispersive X-ray fluorescence (EDXRF) analysis. The relative content of Na and Cl reached 97.87%. Thus, the crystals prepared through solar salt making, with their high NaCl content, have potential value for use as industrial salts. During the membrane filtration test on the separation and desalination device for farmland saline–alkaline water, the bivalent cations Ca^2+^ and Mg^2+^ were not completely removed, mainly owing to the unstable working pressures of the membranes. In this sense, stabilizing the working pressures of membranes in subsequent process optimization will improve the ability of the membranes to remove bivalent cations from saline–alkaline water.

## 5. Discussion

### 5.1. Effect of the Separation and Desalination Device for Farmland Saline–Alkaline Water

This pretreatment device can effectively extend the service life of the composite NF–RO membrane system in arid areas, protecting it from mechanical damage. When the farmland saline–alkaline water was pretreated by the device, the water yielded had a turbidity of <1 NTU and an SDI of <3, which met the requirements of the filter unit for inlet water quality. When the water yielded by the pretreatment device was led into the filter unit and filtered by the composite NF–RO membrane system, the water yielded conformed to the Standard for Irrigation Water Quality (GB 5084-2021) [32]. The total desalination rate (96%) satisfied the process demands of the front-end technology of the separation and desalination device for farmland saline–alkaline water. The water yielded by the filter unit flowed into the circulating solar collector tube and continuously absorbed thermal energy from the inner wall of the circulating solar collector tube until it boiled. The most important factors that influenced the flash efficiency were the vacuum degree of the flash tank under the action of the water-ring vacuum pump and the external heat source of the circulating solar collector tube. A higher temperature of the concentrated water in the circulating solar collector tube improved the gas–liquid separation effect of the flash tank and the flash efficiency. Under the action of the water-ring vacuum pump, the condensation efficiency of the shell-and-tube circulating condenser increased with increasing vacuum degree. In summary, the design of key parts can meet the working requirements of the separation and desalination device for farmland saline–alkaline water in arid areas.

### 5.2. Comparative Analysis of the Separation and Desalination Device for Farmland Saline–Alkaline Water

Owing to differences in process technologies, the separation and desalination device for farmland saline–alkaline water proposed in this study differs from the existing brackish water and seawater desalination devices in terms of energy consumption, water yield, and cost. (1) The latent heat of evaporation in solar stills [35] fails to be fully utilized, which causes considerable heat losses. Solar stills have simple processes, but they occupy a large floor area. (2) Membrane filter units [36] are highly prone to membrane fouling, which affects their working efficiency, increases the frequency of membrane replacement, and increases the cost of the whole device. (3) Multi-stage flash [37] faces the problems of high energy demand, a large investment, serious corrosion, and slow startup. In contrast, the separation and desalination device proposed in this study not only controls the components of crystals and saves water resources, but also generates agricultural irrigation water without a chemical treatment. In this way, the device realizes the reutilization of water resources and effectively solves the problems of agricultural irrigation water and soil salination faced by arid areas.

### 5.3. Application Prospect of Saline–Alkali Water Separation and Desalination Device in an Arid Area

Here, we take Xinjiang as an example. According to an investigation by the Agricultural Department [38], salinization of soil reduces the output of 720 million kg of grain and 130,500 carloads of cotton per year in Xinjiang, resulting in an economic loss of RMB 3.5 billion. The design of the saline water separation and desalination device for the improvement of soil salinization in croplands in dry areas d provides a theoretical basis and support for agricultural and economic development; in the field of saline water treatment, such equipment is a relatively new technology, and it can effectively reduce the salinity, turbidity, and total hardness of water in croplands, thus solving the problem of shortages of freshwater resources. The saline–alkali water separation and desalination device can treat the saline–alkali water in the drainage channel of a cropland and make it into agricultural irrigation water that can be reused, thus providing a technical reference for shortages of agricultural water in arid areas. Solar photovoltaic power generation is used to provide electricity for the cropland saline–alkali water separation and desalination device; therefore, the device can also be applied in remote rural areas that are arid in order to improve local water conditions and the quality of life. To sum up, this saline–alkali water separation and desalination device has great development potential and application prospects in arid farmlands.

## 6. Conclusions

The authors of this study designed a separation and desalination device for farmland saline–alkaline water that is suitable for arid areas. Based on experimental validations, the following conclusions were drawn: After pretreatment, the total salinity of the saline–alkaline water was reduced by 4.9% and had a turbidity of <1 NTU and an SDI of <3. The separation and desalination device effectively protected the composite NF–RO membrane system, extended the service life of the membranes, and met the working conditions of the composite NF–RO membrane system. When the composite NF–RO membrane system was used to filter saline–alkaline water, it achieved a desalination rate of 97.06%, a total hardness removal rate of 97.83%, and a Cl^−^ removal rate of 99.65%.

The final water yielded had a mean salinity of 631.66 mg/L, a mean total hardness of 12.8 mg/L, and a mean Cl^−^ content of 50.79 mg/L, which conformed to the Standard for Irrigation Water Quality (GB 5084-2021) and enabled the reutilization of water resources. The design of horizontal series connections for the circulating solar collector tube effectively increased the working efficiency of the collector tube. The concentrated water in the collector tube was continuously heated until boiling. Under the test conditions, the collection area of the circulating solar collector tube could basically meet the heat demand of the flash tank for distillation. The freshwater yield of the flash tank peaked when the vacuum degree of the flash tank was 0.8 bar and the inlet temperature of the concentrated water was 80 °C. The designed length and number of radiating tubes for the shell-and-tube circulating condenser met the requirements of vapor condensation. The crystals obtained from the production of solar salt contained a large content of NaCl, suggesting that the precipitates have potential value as industrial salts and that the wastes could be reutilized.

## Figures and Tables

**Table 1:** Composition of saline–alkali water after pretreatment.

Ionic Composition of Saline–Alkali Water	Ion Mass Concentration (mg/L)	Percentage of Ion Mass Concentration (%)
Na^+^	4411.58	27.38
Ca^2+^	798.75	4.96
Mg^2+^	543.41	3.37
Cl^−^	7468.89	46.36
SO_4_^2−^	2124.12	13.19
HCO_3_^−^	763.25	4.74
Total salt	16110	100

**Table 2:** Change in the quality of the saline–alkali water.

Index	Raw Salt Alkaline Water	Saline–Alkaline Water after Pretreatment
Average salt content (mg/L)	16,834	16,110
Average turbidity (NTU)	6.9	<1
Average pollution index (SDI)	6.3	<3

**Table 3:** Filtration effects of the nanofiltration and reverse osmosis membranes.

Test Items		Desalination Rate (%)	Hardness Removal Rate (%)	Cl^−^ Removal Rate (%)
ROmembrane	Primary RO water production	27.63	97.23	75.81
	Secondary RO water production	65.36	95.38	91.03
	Tertiary RO water production	93.01	96.69	96.74
NFmembrane	Primary NF water production	31.29	94.83	79.25
	Secondary NF water production	67.48	95.96	90.47
	Tertiary NF water production	94.83	97.94	96.83
NF–ROmembrane	Primary NF–RO water production	28.81	89.62	76.81
	Primary NF–RO water production	66.01	94.65	89.74
	Primary NF–RO water production	97.06	97.83	99.65

**Table 4:** Crystal precipitation effect.

Time (d)	1	2	3	4	5	6	7	8
Theoretical crystallization weight (a)	7.26	6.89	6.94	7.17	7.03	7.14	7.09	7.28
Actual crystallization weight (b)	6.97	6.65	6.81	6.98	6.84	6.83	6.85	7.06
Crystallization rate (b/a)	96.01%	96.52%	98.15%	97.35%	97.29%	95.65%	96.61%	96.97%

**Table 5:** Crystal composition analysis.

Element	Na	Ca	Mg	Cl
Relative content of elements (%)	46.12	1.36	0.77	51.75

## Data Availability

The data presented in this study are available on request from the corresponding author.
